# A Systematic Review of Treatment Options and Clinical Outcomes in Pemphigoid Gestationis

**DOI:** 10.3389/fmed.2020.604945

**Published:** 2020-11-20

**Authors:** Giovanni Genovese, Federica Derlino, Amilcare Cerri, Chiara Moltrasio, Simona Muratori, Emilio Berti, Angelo Valerio Marzano

**Affiliations:** ^1^Dermatology Unit, Fondazione IRCCS Ca' Granda Ospedale Maggiore Policlinico, Milan, Italy; ^2^Department of Pathophysiology and Transplantation, Università degli Studi di Milano, Milan, Italy; ^3^Dermatology Unit, ASST Santi Paolo e Carlo, Milan, Italy; ^4^Department of Health Sciences, Università degli Studi di Milano, Milan, Italy

**Keywords:** pemphigoid gestationis, autoimmune bullous diseases, herpes gestationis, pregnancy, systematic review, treatment

## Abstract

**Background:** Treatment regimens for pemphigoid gestationis (PG) are non-standardized, with most evidence derived from individual case reports or small series.

**Objectives:** To systematically review current literature on treatments and clinical outcomes of PG and to establish recommendations on its therapeutic management.

**Methods:** An a priori protocol was designed based on PRISMA guidelines. PubMed, Scopus, and Web of Science databases were searched for English-language articles detailing PG treatments and clinical outcomes, published between 1970 and March 2020.

**Results:** In total, 109 articles including 140 PG patients were analyzed. No randomized controlled trials or robust observational studies detailing PG treatment were found. Systemic corticosteroids ± topical corticosteroids and/or antihistamines were the most frequently prescribed treatment modality (*n* = 74/137; 54%). Complete remission was achieved by 114/136 (83.8%) patients. Sixty-four patients (45.7%) were given more than one treatment modality due to side effects or ineffectiveness. Leaving aside topical corticosteroids as monotherapy ± antihistamines in patients with mild disease, systemic corticosteroids ± topical corticosteroids and/or antihistamines led to complete remission in the highest proportion of patients (83%), while steroid-sparing treatments ± topical corticosteroids and/or antihistamines were associated with the lowest proportion of flares (55.5%).

**Limitations:** The review has been drafted based on a limited number of single case reports and small case series. Underreporting/underdiagnosis of patients with mild-to-moderate PG, partial/absent follow-up, absence of precise description of neonatal outcomes and lack of validated objective scores for measuring disease severity are other limitations of our study. Our systematic review was affected by publication bias.

**Conclusion:** Systemic corticosteroids are the most frequently used treatment for PG. Whilst most patients achieve complete remission, many of them have refractory/persistent disease requiring multiple lines of therapy. Therefore, we provided an algorithm for PG treatment integrating the results of this systematic review with current knowledge available for bullous pemphigoid. High-quality studies will further help assess the effectiveness of different treatment options for PG.

## Introduction

Pemphigoid gestationis (PG), formerly known as herpes gestationis, is a rare subepidermal autoimmune blistering disease belonging to the group of specific dermatoses of pregnancy (1). It is characterized by intensely pruritic urticarial plaques and/or vesiculobullous lesions typically starting in the periumbilical region. Although PG usually occurs in late pregnancy or puerperium, it may also be rarely associated with gestational trophoblastic disease (GTD), including choriocarcinoma and hydatiform mole (2). Linear deposits of complement fraction 3 (C3) ± immunoglobulin (Ig) G along the dermal-epidermal junction on direct immunofluorescence (DIF) of perilesional skin are mandatory to confirm the clinical suspicion (3). PG may have a chronic-relapsing course, with flares usually occurring after delivery, during menses, or in association with the use of hormonal contraceptives. Recurrences in subsequent pregnancies are common (4). Systemic and topical corticosteroids are empirically recognized as a cornerstone of PG treatment, especially during the gestation period and in mild-to-moderate cases. On the other hand, a wide variety of therapeutic approaches, including steroid-sparing agents such as intravenous immunoglobulin therapy, azathioprine, and dapsone, have been reported for persistent cases refractory to first-line regimens or patients with intolerance to (or medical inadvisability of) systemic corticosteroids. Moreover, the management of this disease may be challenging owing to the safety concerns during pregnancy or lactation of some immunosuppressive drugs (5). Nevertheless, except for the recommendations of the French Society of Dermatology (6), no specific guidelines have been developed for the treatment of PG. In addition, no systematic review has been carried out on therapeutic options for PG to date and the current data available on PG treatment are largely based on case reports and case series. This systematic review aimed at providing a comprehensive and up-to-date analysis of treatment options employed for PG and developing a therapeutic algorithm for this disease.

## Materials and Methods

### Protocol and Literature Search

The recommendations contained in the Preferred Reporting Items for Systematic Reviews and Meta-Analyses (PRISMA) statement (7) were followed. The literature review was conducted using PubMed, Scopus and Web of Science databases. The search strings were the following: “pemphigoid AND gestationis” and “herpes AND gestationis.” Publications between 1 January 1970 and 24 March 2020 were searched independently and cross-checked by two researchers (GG and DF) (Supplementary Table 1).

### Selection of Articles

Articles were screened by title and abstract and those deemed relevant were reviewed in full text. Any disagreements regarding article suitability were solved by a third independent author (AVM). The articles were included in the qualitative synthesis if (i) PG diagnosis was based on a clinical picture suggestive of subepithelial autoimmune bullous disease occurring during pregnancy, post-partum or in association with GTD, a histopathologic image of subepidermal detachment and a DIF test showing linear deposition of C3 ± IgG along the dermal-epidermal junction, (ii) they were published in English, (iii) they documented in detail the treatment and clinical outcome.

### Outcomes

Primary outcome measures were different treatment regimens and response to therapy, defined as complete remission (CR), partial response (PR), and active disease at the end of follow-up. Secondary outcome measures were side effects, occurrence of flares, and pregnancy-related outcomes in mothers and children.

### Data Extraction and Analysis

Two authors (GG and FD) critically reviewed the included articles and independently extracted the following variables onto a Microsoft Excel spreadsheet: age at onset, age at main treatment initiation, gestational age at PG onset, gestational age at first treatment initiation, number of pregnancies, PG recurrence in different pregnancies, first treatment initiation, first treatment, main treatment initiation, main treatment, lines of therapy, modality of treatment, prednisone-equivalent dose of systemic corticosteroids (initial and maximum), maternal disease outcome, gestational age at delivery, mode of delivery, occurrence of flare(s), cause of flare(s), persistent course, follow-up duration, side effects, and newborn outcome. Relevant data were not available for every patient; thus, percentages refer to the total number of patients for whom information regarding a specific outcome was available or could be concluded.

The main treatment was defined as the treatment associated with patient clinical outcome at the end of follow-up. In patients who underwent only one line of therapy, main treatment coincided with first-line treatment. Conversely, in patients who underwent more than one line of therapy, treatments which had been given before main treatment were referred to as first-line treatments. The following modalities of treatment were recognized: (i) systemic corticosteroids ± topical corticosteroids and/or antihistamines; (ii) systemic corticosteroids combined with steroid-sparing treatments ± topical corticosteroids and/or antihistamines; (iii) steroid-sparing treatments ± topical corticosteroids and/or antihistamines; (iv) topical corticosteroids as monotherapy ± antihistamines. Steroid-sparing treatments included all the treatments that were given in addition to or instead of systemic corticosteroids to lower the dose of systemic corticosteroids needed or avoid using them. CR, defined as the absence of lesions at the end of follow-up, was subdivided into off-therapy or on-therapy. Simplified definitions of CR on-therapy and CR off-therapy were adapted from the definitions and outcome measures for bullous pemphigoid recommended by an international panel of experts (8) CR off-therapy was defined as an absence of new or established lesions or pruritic symptoms while the patient is off all PG therapy, while CR on-therapy was defined as the absence of new or established lesions or pruritus while the patient is receiving any topical or systemic treatment (8). PR was defined as improvement without complete healing of skin lesions, while active disease as absence of improvement/worsening of disease. A flare was defined as the appearance of new lesions or the extension of established lesions in a patient who had previously achieved CR. Regarding the therapeutic algorithm for PG management, mild, and moderate-to-severe PG were defined according to the classification adopted in the European Dermatology Forum/European Academy of Dermatology and Venereology guidelines on bullous pemphigoid management (9). In patients with onset during pregnancy or GTD, the course was defined as persistent if the disease was still active ≥6 months after delivery/abortion/GTD remission. In patients with post-partum onset, the course was regarded as persistent in case of disease activity lasting ≥ 6months from disease onset (10). Categorical variables were reported as frequencies and percentages while continuous variables were reported medians and interquartile range (IQR) or means and standard deviation (SD). Percentages refer to the number of patients for whom information about a specific parameter was available or inferable. The statistical software SAS (release 9.4, SAS Institute, Inc., Cary, North Carolina) was used to perform all the statistical analyses.

### Quality and Risk of Bias

Two authors (GG and FD) assessed the methodological quality of the evidence and risk of bias of the included studies independently. Any disagreement was resolved through discussion with a third author (AVM).

## Results

### Identification of Eligible Articles

**Figure 1:**
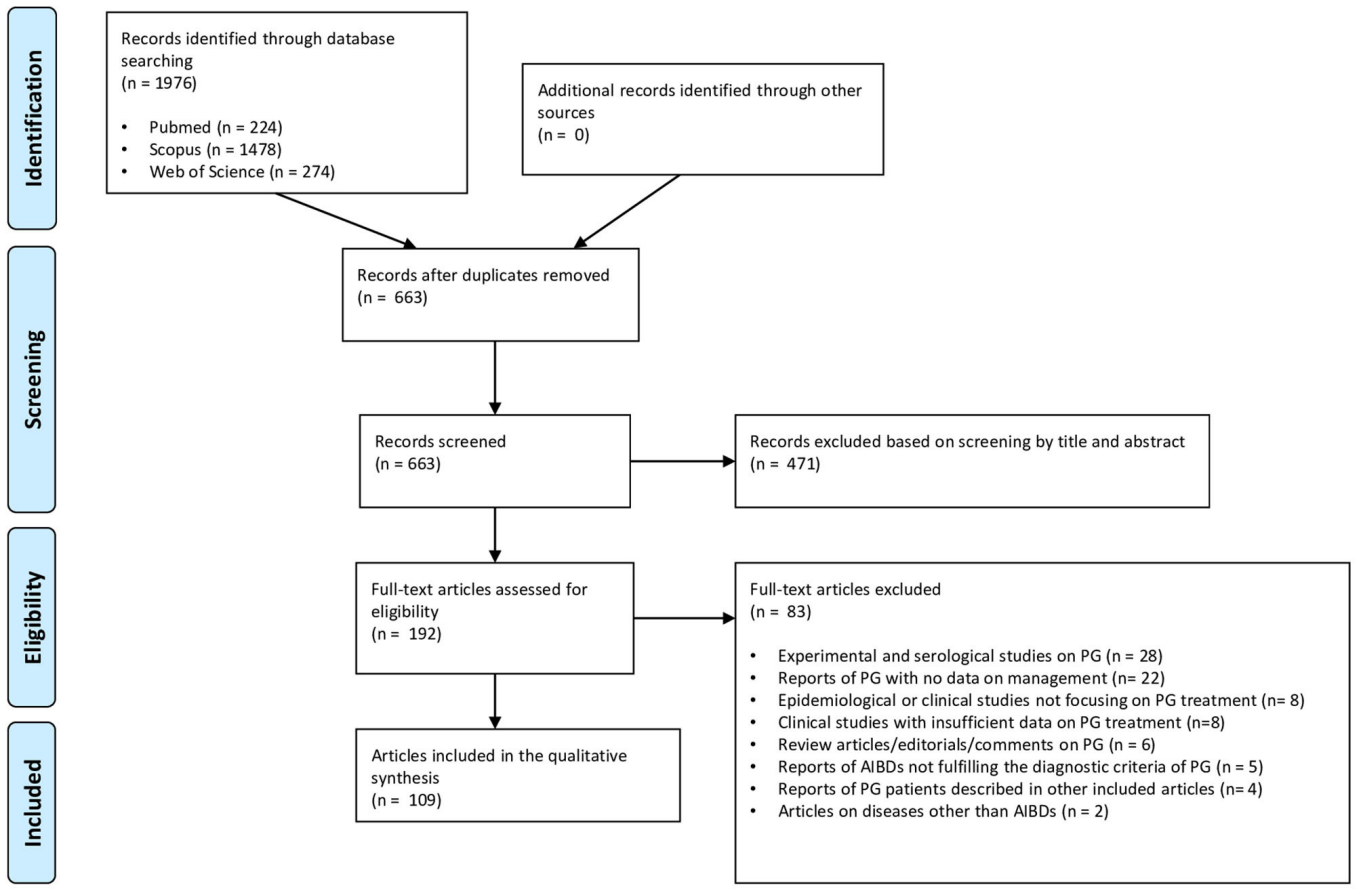
PRISMA flow diagram detailing literature search and study selection process for systematic review.

As shown in the PRISMA flow diagram (Figure 1), the literature search identified 1976 references. After exclusion of duplicates (*n* = 1313) and manuscripts deemed irrelevant based on title and/or abstract screening or written in languages other than English (*n* = 471), the remaining 192 articles were reviewed in full text. Eventually, 109 articles, (96 individual case reports and 13 case series), met the eligibility criteria and were included in the qualitative synthesis (Supplementary Table 2). A final sample of 140 PG patients from 29 different countries was analyzed. Geographical areas of the reports were subdivided as follows: Europe (*n* = 48); North America (*n* = 25); East Asia (*n* = 15); West Asia (*n* = 7); South Asia (*n* = 6); South America (*n* = 3); Australia (*n* = 3); North Africa (*n* = 1); Southeastern Asia (*n* = 1).

### Demographic and Clinical Features

Demographic and clinical data are summarized in Table 1. Median age at onset was 28 (IQR: 25-33) years, while the median age at main treatment initiation was 30 (IQR: 26-34) years. PG was associated at onset with pregnancy in 85% (*n* = 119) of cases, whereas it occurred during the post-partum period in 15 (10.7%) patients and in association with GTD in six (4.2%) patients. In particular, two pregnancies followed egg donation and one pregnancy was ectopic (tubal). Median gestational age at PG onset was 7 (5-8) months. Twenty-nine (41.4%) out of 70 multigravida patients with available data experienced PG recurrence in different pregnancies, while in the remaining 41 (58.6%) PG did not recur. On the other hand, 63/132 (45.7%) patients with available data were primigravida.

**Table 1:** Demographic and clinical features of the 140 patients with pemphigoid gestationis included in the study.

			**Reported patients**
Age at onset, years, median (IQR)		28 (25-33)	123
Gestational age at pemphigoid gestationis onset, months, median (IQR)		7 (5-8)	108
Condition associated with pemphigoid gestationis onset, *n* (%)	Pregnancy^*^	119 (85)	140
	Post-partum	15 (10.7)	140
	Hydatiform mole	3 (2.1)	140
	Choriocarcinoma	3 (2.1)	140
Age at main treatment initiation, years, median (IQR)		30 (26-34)	129
Gestational age at first treatment initiation, months, median (IQR)^**^		7 (5-8)	89
Multigravida patients, *n* (%)		69 (52.3)	132
Primigravida patients, *n* (%)		63 (47.7)	132
Median number of pregnancies, median (IQR)		2 (1-3)	128
Recurrence in different pregnancies in multigravida patients^***^	Recurrent, *n* (%)	29 (41.4)	70
	Not recurrent, *n* (%)	41 (58.6)	70

*IQR, interquartile range*.

* *One pregnancy was ectopic (tubal), while two pregnancies were achieved after ovodonation*.

** *In 24 patients, first treatment was started after delivery/abortion; furthermore, patients with gestational trophoblastic disease or ectopic pregnancy (n = 7) and patients who received no treatment (n = 3) were not included in the analysis*.

*** *Primigravida patients (n = 63) were not included in the analysis*.

### Treatment Strategies

Systemic corticosteroids ± topical corticosteroids and/or antihistamines were the most frequent main treatment modality (*n* = 74/137; 54%), being followed by systemic corticosteroids combined with steroid-sparing treatments ± topical corticosteroids and/or antihistamines (*n* = 35/137; 25.6%), steroid-sparing treatments ± topical corticosteroids and/or antihistamines (*n* = 18/137; 13.1%) and topical corticosteroids as monotherapy ± antihistamines (*n* = 19/137;7.3%) (Table 2). Three patients received no treatment. Among systemic corticosteroids, the most frequently administered was prednisone (*n* = 47/109; 43.1%), followed by prednisolone (*n* = 39/109; 35.8%), betamethasone (*n* = 8/109; 7.3%), methylprednisolone (*n* = 6/109; 5.5%), dexamethasone (*n* = 3/109; 2.8%), and fluocortolone (*n* = 2; 2.8%). The mean initial daily prednisone-equivalent dosage of systemic corticosteroids—specified in 96/115 patients treated with these agents—corresponded to 52.8 mg/day, while the mean maximum prednisone-equivalent dosage of systemic corticosteroids—specified in 93/114 patients—was 71.9 mg/day. Intravenous immunoglobulin therapy was the most commonly used steroid-sparing treatment (*n* = 12/54; 22.2%), followed by azathioprine (*n* = 8/54; 14.8%), dapsone (*n* = 7/54; 13%), cyclosporine (*n* = 6/54; 11.1%), and pyridoxine (*n* = 5/54; 9.3%). Treatments given to ≤ 3 patients included plasmapheresis/plasma exchange, minocycline, nicotinamide, immunoadsorption, surgery ± chemotherapy ± radiotherapy for choriocarcinoma, rituximab, ritodrine, doxycycline, erythromycin, cyclophosphamide, methotrexate, sulfapyridine, chemical oophorectomy with goserelin, surgical oophorectomy + hysterectomy, mycophenolate mofetil, roxithromycin. First-line treatment was initiated during pregnancy in 99/125 (79.2%) patients and after delivery/abortion in 26/125 (20.8%) patients (Table 3). Main treatment was started during pregnancy in 76/128 (59.4%) cases and after delivery/abortion in 52/128 (40.6%) cases. Spontaneous CR or PR was achieved without/regardless of treatment in five (3.6%) patients.

**Table 2:** Modalities of main treatment and specific therapy of the included patients with pemphigoid gestationis.

			**Reported patients**
Modalities of main treatment, *n* (%)^*^	Systemic corticosteroids ± topical corticosteroids and/or antihistamines	74 (54)	137
	Systemic corticosteroids combined with steroid-sparing treatments ± topical corticosteroids and/or antihistamines	35 (25.6)	137
	Steroid-sparing treatments ± topical corticosteroids and/or antihistamines	18 (13.1)	137
	Topical corticosteroids as monotherapy ± antihistamines	10 (7.3)	137
Systemic corticosteroids, *n* (%)	Prednisone	47 (43.1)	109
	Prednisolone	39 (35.8)	109
	Betamethasone	8 (7.3)	109
	Methylprednisolone	6 (5.5)	109
	Dexamethasone	3 (2.8)	109
	Fluocortolone	3 (2.8)	109
	Systemic corticosteroids not specified	5 (4.6)	109
Prednisone-equivalent dose of systemic corticosteroids, mg/day (SD)	Mean initial dose	52.8 (125)	96
	Mean maximum dose	71.9 (139.6)	93
Steroid-sparing treatments, *n* (%)	Intravenous immunoglobulin therapy	12 (22.2)	54
	Azathioprine	8 (14.8)	54
	Dapsone	7 (13)	54
	Cyclosporine	6 (11.1)	54
	Pyridoxine	5 (9.3)	54
	Plasmapheresis/plasma exchange	3 (5.6)	54
	Minocycline	3 (5.6)	54
	Nicotinamide	3 (5.6)	54
	Immunoadsorption	3 (5.6)	54
	Treatment of choriocarcinoma (surgery ± chemotherapy ± radiotherapy)	2 (3.7)	54
	Rituximab	2 (3.7)	54
	Ritodrine	2 (3.7)	54
	Doxycycline	2 (3.7)	54
	Erythromycin	2 (3.7)	54
	Cyclophosphamide	2 (3.7)	54
	Methotrexate	2 (3.7)	54
	Sulfapyridine	1 (1.9)	54
	Chemical oophorectomy (goserelin)	1 (1.9)	54
	Surgical oophorectomy + hysterectomy	1 (1.9)	54
	Mycophenolate mofetil	1 (1.9)	54
	Roxithromycin	1 (1.9)	54

*SD, standard deviation*.

* *Three patients out of 140 received no treatment*.

**Table 3:** Lines of therapy stratified by clinical outcome and treatment starting of the included patients with pemphigoid gestationis.

		***n* (%)**	**Reported patients**
Spontaneous achievement of CR/PR without/regardless of treatment		5 (3.6)	140
Only one treatment modality	CR or PR^*^	66 (47.1)	140
	Active disease^*^	2 (1.4)	140
More than one treatment modality	CR or PR	58 (41.4)	140
	Active disease	6 (4.3)	140
First-line treatment initiation	During pregnancy^**^	99 (79.2)	125
	After delivery/abortion^**^	26 (20.8)	125
Main treatment initiation	During pregnancy^**^	76 (59.4)	128
	After delivery/abortion^**^	52 (40.6)	128

*CR, complete remission; PR, partial response*.

* *Clinical outcome was not available for three patients who were treated with only one treatment modality*.

** *Patients with gestational trophoblastic disease or ectopic pregnancy (n = 7) and patients who received no treatment (n = 3) were not included in the analysis*.

### Maternal Clinical Outcomes

In the 74 patients with available data, the median follow-up period lasted 9 (IQR: 5-18.3) months (Table 4). Clinical outcome was available in 136 patients: among them, CR was achieved by 114 (83.8%) patients, of whom 70 (51.5%) had CR off-therapy, 14 (10.3%) had CR on-therapy and four (2.9%) had spontaneous CR. For the remaining 26 (19.1%) patients with CR, it could not be assessed whether treatment was still ongoing at the end of follow-up period. At the end of follow-up, PR was achieved by 14 (10.3%) patients, while eight (5.9%) still had active disease. Of 68/140 (48.6%) patients treated with only one treatment, 66 (97.1%) achieved CR or PR; of 64/140 (45.7%) patients treated with more than one treatment modality, 58 (90.6%) experienced CR or PR (Table 3). As shown in Table 4, a persistent course was observed in 32/135 (23.7%) cases and flares were observed in 83/137 (60.6%) patients. The latter ones were mainly linked to the post-partum/post-abortion period (*n* = 42/83; 50.6%) but were also linked to poor response to therapy, corticosteroid tapering, menstruation, hormonal contraception/hormonal therapy, treatment withdrawal. Median gestational age at delivery—specified in 67 patients—was 37.5 (IQR: 35-39) weeks. Mode of delivery, reported in 60/133 pregnant women, was elective cesarean section in 25 (41.7%) patients, spontaneous vaginal delivery in 21 (35%), emergency cesarean section in eight (6%), and vaginal delivery after labor induction in seven (5.3%). Intrauterine spontaneous fetal death was observed in 8/133 (5.3%) cases, while voluntary abortion and stillbirth were observed in one patient each.

**Table 4:** Maternal and newborn clinical outcomes of included cases with pemphigoid gestationis.

			**Reported patients**
Follow-up period, months, median (IQR)		9 (5-18.3)	74
Maternal disease outcome, *n* (%)	Complete remission^*^	26 (19.1)	136
	Complete remission on-therapy	14 (10.3)	136
	Complete remission off-therapy	70 (51.5)	136
	Complete remission (spontaneous)	4 (2.9)	136
	Partial remission	14 (10.3)	136
	Active disease at the end of follow-up	8 (5.9)	136
Persistent course, *n* (%)		32 (23.7)	135
Flares, *n* (%)		83 (60.6)	137
Cause of flares^**^	After delivery/abortion/gestational trophoblastic disease remission	42 (50.6)	83
	Poor response to therapy	20 (24.1)	83
	Corticosteroid tapering	18 (21.7)	83
	Menstruation	13 (15.7)	83
	Hormonal contraception/hormonal therapy	7 (8.4)	83
	Treatment withdrawal	5 (6)	83
Gestational age at delivery, weeks, median (IQR)		37.8 (35-39)	66
Pre-term births (<37 gestational weeks), *n* (%)		23 (34.8)	66
Mode of delivery, *n* (%)^***^	Elective cesarean section	25 (41.7)	60
	Spontaneous vaginal delivery	21 (35)	60
	Emergency cesarean section	8 (13.3)	60
	Vaginal delivery after labor induction	6 (10)	60
Intrauterine fetal death/ stillbirth, *n* (%)	Spontaneous abortion	7 (77.8)	9
	Voluntary abortion	1 (11.1)	9
	Stillbirth	1 (11.1)	9
Live-born children, *n* (%)^***^	Healthy without skin lesions^****^	83 (83)	100
	Healthy with skin lesions	13 (13)	100
	Postnatal death due to sepsis	2 (2)	100
	Severe growth retardation	2 (2)	100

* *The concomitant status of treatment could not be concluded in 26 patients who achieved complete remission*.

** *More than one cause of flare was scored in 20 patients*.

*** *Patients with gestational trophoblastic disease or ectopic pregnancy (n = 7) as well as intrauterine fetal deaths or stillbirths (n = 9) were not included in the analysis*.

**** *One patient had a twin pregnancy*.

### Newborn Clinical Outcomes

Of 100 live-born children with available data, 83 (83%) were healthy without skin lesions, while 13 (13%) had skin lesions (urticarial and/or bullous). Two babies born from pregnancies complicated by anhydramnios died of sepsis few days after delivery and two had severe growth retardation (Table 4).

### Maternal Disease Outcomes Stratified by Treatment Modality

As shown in Table 5, CR was achieved by 59/74 (79.7%) patients who received systemic corticosteroids ± topical corticosteroids and/or antihistamines, by 30/35 (85.7%) patients who received systemic corticosteroids combined with steroid-sparing agents ± topical corticosteroids and/or antihistamines, by 12/18 (66.7%) patients who received steroid-sparing treatments ± topical corticosteroids and/or antihistamines and by 9/10 (90%) patients who received topical corticosteroids as monotherapy ± antihistamines. Flares were observed in 46/74 (62.2%) patients treated with systemic corticosteroids ± topical corticosteroids and/or antihistamines, in 25/35 (71.4%) patients treated with systemic corticosteroids combined with steroid-sparing treatments ± topical corticosteroids and/or antihistamines, in 10/18 (55.5%) patients treated with steroid-sparing treatments ± topical corticosteroids and/or antihistamines and in 2/10 (20%) patients treated with topical corticosteroids as monotherapy ± antihistamines.

**Table 5:** Clinical maternal outcomes of included patients with pemphigoid gestationis divided according to treatment modality.

**Treatment modality**	**Complete remission, *n* (%)**	**Complete remission on-therapy, *n* (%)**	**Complete remission off-therapy, *n* (%)**	**Complete remission (spontaneous), *n* (%)**	**Partial response, *n* (%)**	**Active disease, *n* (%)**	**At least one flare during follow-up, *n* (%)**
Systemic corticosteroids ± topical corticosteroids and/or antihistamines (*n* = 74)^*^	15 (21.1)	4 (5.6)	40 (56.3)	0	9 (12.7)	3 (4.2)	46 (65.8)
Systemic corticosteroids combined with steroid-sparing agents ± topical corticosteroids and/or antihistamines, (*n* = 35)	7 (20)	8 (22.9)	15 (42.9)	0	1 (2.9)	3 (8.6)	25 (71.4)
Steroid-sparing treatments ± topical corticosteroids and/or antihistamines, (*n* = 18)	1 (5.6)	2 (11.1)	9 (50)	1 (5.6)	3 (16.7)	2 (11.1)	10 (55.5)
Topical corticosteroids as monotherapy ± antihistamines, (*n* = 10)	3 (30)	0	6 (60)	1 (10)	0	0	2 (20)

* *Data on clinical outcome of three out of 74 patients treated with systemic corticosteroids +/− topical corticosteroids and/or oral antihistamines were not available*.

### Newborn Clinical Outcomes Stratified by Treatment Modality

Newborn outcome clinical data were available for 61 deliveries that had main treatment initiation during pregnancy. Among the 48 pregnant patients that had healthy newborns without skin lesions, main treatment consisted of systemic corticosteroids ± topical corticosteroids and/or antihistamines in 30 (62.5%) cases, topical corticosteroids as monotherapy ± antihistamines in 3 (6.25%) cases, steroid-sparing treatments ± topical corticosteroids and/or antihistamines in 6 (12.5%) cases and systemic corticosteroids combined with steroid-sparing agents ± topical corticosteroids and/or antihistamines in 9 (18.75%) cases. Among the 9 pregnant patients that had healthy newborns with skin lesions, main treatment consisted of systemic corticosteroids ± topical corticosteroids and/or antihistamines in 6 (66.7%) cases, topical corticosteroids as monotherapy ± antihistamines in 1 (11.1%) cases, steroid-sparing treatments ± topical corticosteroids and/or antihistamines in 2 (22.2%) cases. All 3 patients whose newborns developed complications such oligohydramnios or growth retardation and the only patient that experienced intrauterine fetal death had been treated with systemic corticosteroids ± topical corticosteroids and/or antihistamines.

### Side Effects

Most frequently reported side effects were attributed to corticosteroids (iatrogenic Cushing syndrome [*n* = 9], steroidal diabetes [*n* = 5], arterial hypertension [*n* = 3], steroid myopathy [*n* = 1], osteoporosis [*n* = 1], vaginal bleeding [*n* = 1], mood changes [*n* = 1], striae distensae [*n* = 1], fetal macrosomia and polyhydramnios [*n* = 1]). Other infrequent side effects were ascribed to minocycline (dizziness [*n* = 1]), goserelin (flushing [*n* = 1]), intramuscular gold (proteinuria [*n* = 1]), azathioprine (elevated liver enzymes [*n* = 1]), dapsone (skin toxicity [*n* = 1], malaise [*n* = 1]), sulfapyridine (skin toxicity [*n* = 1]), cyclosporine (elevated creatinine levels [*n* = 1]), intravenous immunoglobulin (headache [*n* = 1]).

## Discussion

This systematic review, the first one focusing on PG, analyzed the current literature on PG therapeutic strategies and clinical outcomes, intending to provide accurate insights into its characteristics and help cope with the choice of the best treatment during pregnancy or lactation. Due to the lack of randomized or controlled trials, we collected 140 cases of PG extracted from case reports and small case series.

### Bias and Quality Assessment of the Literature on Pemphigoid Gestationis Treatment

The review has been drafted based on single case reports and small case series, part of which sometimes describing clinical outcomes and treatments only succinctly and omitting drug dosages, particularly dosage per body weight of systemic corticosteroids. The limited number of studies available about this rare disease is another weakness of our systematic review. Patients with mild to moderate PG might have been missed either because they have not been reported or not even been diagnosed. Moreover, follow-up was partial or lacking in most cases. The eight studies excluded from the qualitative synthesis due to lack of detailed information about treatments and clinical outcomes have been reported in Supplementary Table 3 and show results, albeit fragmentary, in line with those highlighted by our analysis. In the reviewed literature, neonatal outcome was almost always mentioned, even though with only occasional reports of neonatal weight or systemic complications bound to maternal treatments. Validated objective scores for measuring severity of illness and outcomes were not used in any study, thus hampering a precise comparison. Assessments on precision cannot be done due to the absence of confidence intervals. Eventually, our systematic review may be affected by publication bias: cases that did not show a good response to treatment are less likely to be published and over-reporting of severe cases may have led to an overestimation of the clinical burden of the disease.

### Evidence About Pemphigoid Gestationis Treatment Emerged From the Systematic Review

**Figure 2:**
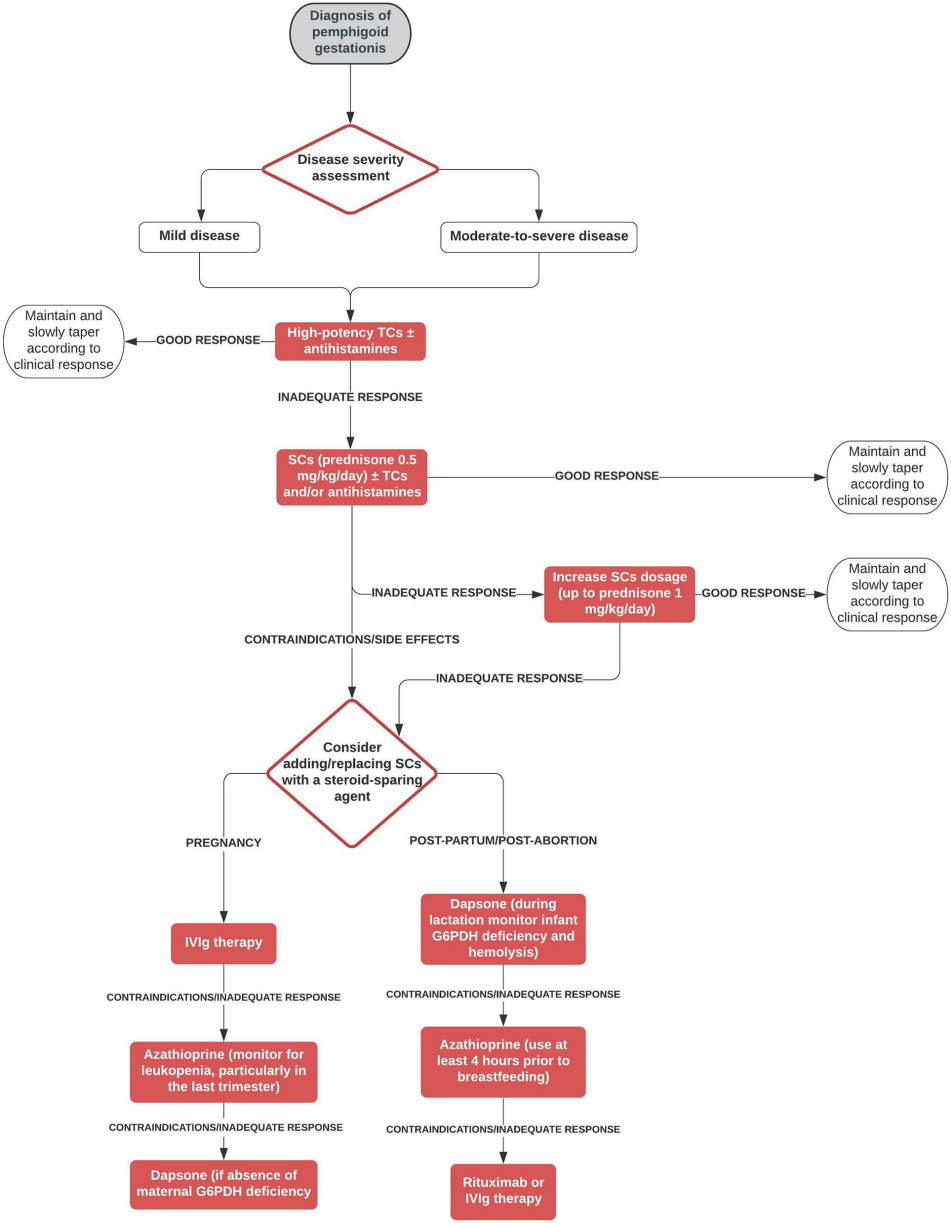
Therapeutic algorithm for pemphigoid gestationis. G6PDH, glucose-6-phosphate dehydrogenase; IVIg, intravenous immunoglobulin; SCs, systemic corticosteroids; TCs, topical corticosteroids.

The most commonly used treatment modality in our cohort was a combination of systemic corticosteroids ± topical corticosteroids and/or antihistamines, with prednisone being the most frequent type of systemic corticosteroid. Mean initial prednisone-equivalent dosage of systemic corticosteroids was nearly 50 mg/day, with mean maximum dosage being approximately 70 mg/day. These clinical data reflect those by Chi et al. (10), who failed to find significant associations between adverse pregnancy outcomes and systemic corticosteroids in their retrospective study on 61 pregnancies, even after stratifying the systemic corticosteroid treatment by the length of treatment or the trimester of beginning treatment. Based on their findings, Chi et al. suggested that the benefit from systemic corticosteroids in patients with PG likely outweighs their potential harm (11). Most patients (83.8%) of our cohort achieved CR. However, a significant proportion (60.6%) of patients had at least one relapse, mainly due to post-partum exacerbation of the disease, and almost 25% of patients showed a persistent course. Interestingly, ~40% of multigravida patients showed a recurrent course in different pregnancies. In our cohort, PG required more than one treatment modality in about 50% of patients and the main treatment was represented by a steroid-sparing agent in addition to systemic corticosteroids in about 25% of patients, thus suggesting a refractory nature of the disease in a great proportion of patients. On the other hand, only 5/140 patients achieved spontaneous remission without/regardless of treatment. Main treatment obviously tended to be started after delivery/abortion in a higher proportion of patients compared with first treatment (40.6 vs. 20.8%, respectively). Topical corticosteroids as monotherapy were prescribed only to a minority (7.3%) of patients and led to a 100% CR rate and low (20%) relapse rate. This favorable course of disease in patients treated with topical corticosteroid monotherapy may be explained by the fact that these patients presented usually with mild disease. Setting aside topical corticosteroids as monotherapy ± antihistamines, systemic corticosteroids ± topical corticosteroids and/or antihistamines was the treatment modality that led to CR in the highest proportion of patients (83%), while steroid-sparing treatments ± topical corticosteroids and/or antihistamines were associated with the highest proportion of absence of flares (45.5%). Intravenous immunoglobulin therapy was the most frequently administered steroid-sparing agent, accounting for 22.2% of the group of steroid-sparing treatments. It was successfully used also as monotherapy (12). The effectiveness and safety of intravenous immunoglobulin therapy in treating pregnant patients with autoimmune bullous diseases have already been demonstrated by Ahmed et al. (13). in a group of patients with pemphigus vulgaris. Azathioprine and dapsone were the second and third most commonly prescribed steroid-sparing medications, always given after delivery/abortion. On the other hand, cyclosporine, which was the fourth most frequently administered steroid-sparing drug, was successfully used also during pregnancy (14, 15). Interestingly, rituximab was used in two persistent cases either during pregnancy to prevent disease flare (16) and after delivery (17). As previously observed in pemphigus, rituximab administered 6–12 months before conception may help prevent disease worsening and achieve stable disease during pregnancy (18, 19). The frequent reports of refractory/persistent cases make the definition of therapeutic approaches for PG an unmet clinical need. Therefore, we propose a decisional algorithm on the basis of both the evidence derived from this systematic review merged with current knowledge available of bullous pemphigoid (9), a subepidermal autoimmune blistering diseases that shares some clinical features with PG (Figure 2).

Safety concerns during pregnancy and lactation have also taken into consideration. In analogy to bullous pemphigoid, high potency topical corticosteroids are proposed as first-choice treatment both in mild disease and in moderate-to-severe PG. Non-fluorinated steroids such as hydrocortisone and prednisolone should be preferred respect to fluorinated steroids such as dexamethasone, methylprednisolone, and betamethasone that are metabolized less extensively by 11β-hydroxysteroid dehydrogenase and easily cross the placental barrier (5). In refractory disease, systemic corticosteroids may be attempted. Even there is proven evidence, oral antihistamines can be used for itch management. In case of failure/contraindication/side effects of systemic corticosteroids, we suggest to consider during pregnancy intravenous immunoglobulin therapy and, as second step, conventional immunosuppressants/immunomodulating agents such as dapsone or azathioprine, which are the last choice, which can be used safely during pregnancy as inferred from many studies involving patients with other diseases treated with these agents (20–23). In the post-partum/post-abortion period, we suggest as first step treatment immunosuppressant/immunomodulating agents, including dapsone and azathioprine, leaving as second choices IVIg and rituximab due to pharmacoeconomic reasons. As far as concerns pregnancy/newborn outcomes, almost 35% of patients experienced a pre-term delivery and elective cesarean section was the most common mode of delivery, being performed in 43% of patients with available data. Fetal distress, placental insufficiency or poor control of the disease may be possible explanations of the latter finding. Among live-born children, only 13 (13%) had skin lesions suggestive of neonatal pemphigoid. Eventually, intrauterine fetal death was observed only in eight cases.

In conclusion, high-potency topical corticosteroids may be recommended as first-line agents either in mild and in moderate-to-severe PG. Systemic corticosteroids may be a useful approach for recalcitrant disease. Intravenous immunoglobulin therapy alone or in combination with systemic corticosteroids may be considered as further line of treatment. Although most patients achieve CR, a considerable proportion of them experience refractory/persistent disease requiring multiple lines of therapy. Randomized controlled trials are needed to better define the effectiveness of different treatments in PG.

## Data Availability Statement

The original contributions presented in the study are included in the article/Supplementary Material, further inquiries can be directed to the corresponding author/s.

## Author Contributions

GG: study design, database management, search strategies, and writing of the manuscript. FD: database management, search strategies, and writing of the manuscript. CM: search strategies. EB, SM, and AC: critical review of the manuscript. AM: study design and critical review of the manuscript. All authors listed have made a substantial contribution to the work and approved it for publication.

## Conflict of Interest

The authors declare that the research was conducted in the absence of any commercial or financial relationships that could be construed as a potential conflict of interest.
