# Supplementary material for: A Systematic Review of Treatment Options and Clinical Outcomes in Pemphigoid Gestationis
Source: Front Med (Lausanne). 2020 Nov 20;7:604945. doi: 10.3389/fmed.2020.604945 (PMC7717454; doi:10.3389/fmed.2020.604945)
Supplement: Supplementary file 1 [file Data_Sheet_1.docx]

**Supplementary table 1.** Literature search strategy

| **Literature search scheme for Pubmed (224 results; 124 for queried terms "pemphigoid” AND gestationis and 100 records for queried terms “herpes” AND gestationis)**  **Search String:** |
| --- |
| \| **#1** \| \| --- \| \| (pemphigoid) AND gestationis AND ((“1970/01/01”[PubDate]: “2020/03/24”[PubDate]))  Query translation: ("pemphigoid, bullous"[MeSH Terms] OR ("pemphigoid"[All Fields] AND "bullous"[All Fields]) OR "bullous pemphigoid"[All Fields] OR "pemphigoid"[All Fields]) AND gestationis[All Fields] AND ("1970/01/01"[PubDate] : "2020/03/24"[PubDate]) \| \| User query: ("pemphigoid, bullous"[MeSH Terms] OR ("pemphigoid"[All Fields] AND "bullous"[All Fields]) OR "bullous pemphigoid"[All Fields] OR "pemphigoid"[All Fields]) AND gestationis[All Fields] AND ("1970/01/01"[PubDate] : "2020/03/24"[PubDate]) \| \| **#2**  "herpes"[All Fields] AND GESTATIONIS [All Fields] AND ("1970/01/01"[PubDate]: "2020/03/24"[PubDate]) \| |
| **Literature search scheme for Scopus (1478 results; 910 for queried terms "pemphigoid” AND gestationis and 568 records for queried terms “herpes” AND gestationis)**  **Search String:** |
| \|  \| **#1**  TITLE-ABS-KEY (''pemphigoid'' AND gestationis) AND PUBYEAR > 1969 AND PUBYEAR < 2021 \| \| --- \| --- \| \|  \| **#2**  TITLE-ABS-KEY (''herpes'' AND gestationis) AND PUBYEAR > 1969 AND PUBYEAR  <  2021 \| |
| **Literature search scheme for Web of Science (274 results; 210 for queried terms "pemphigoid” AND gestationis and 64 records for queried terms “herpes” AND gestationis)**  **Search String:** |
| \|  \| **#1** \| \| --- \| --- \| \|  \| (TS=(pemphigoid* near/2 gestationis*))  *Indexes=SCI-EXPANDED, SSCI, A&HCI, CPCI-S, CPCI-SSH, BKCI-S, BKCI-SSH, ESCI Timespan=1970-2020* \| \|  \| **#2**  (TS=(herpes* near/2 gestationis*))  Indexes: SCI-EXPANDED, SSCI, A&HCI, CPCI-S, CPCI-SSH, BKCI-S, BKCI-SSH, ESCI Timespan=1970-2020 \| |

**Supplementary table 2.** All included publications presenting cases of patients with pemphigoid gestationis

| **Year of publication** | **First author** | **Year of publication** | **First author** | **Year of publication** | **First author** |
| --- | --- | --- | --- | --- | --- |
| 1976 | Hertz *et al.*^1^ | 1996 | Jenkins *et al.*^37^ | 2013 | Matsumoto *et al.*^73^ |
| 1976 | Reid *et al.*^2^ | 1996 | Lowe *et al.*^38^ | 2013 | Podolec-Rubis *et al.*^74^ |
| 1977 | Carruthers *et al.*^3^ | 1997 | Schild *et al.*^39^ | 2014 | Cabral *et al.*^75^ |
| 1977 | Reunala *et al.*^4^ | 1997 | Vaughan Jones *et al.*^40^ | 2014 | Hapa *et al.*^76^ |
| 1978 | Schiffner *et al.*^5^ | 1998 | Hern *et al.*^41^ | 2014 | Henry^77^ |
| 1980 | Grimwood *et al.*^6^ | 1999 | Chen *et al.*^42^ | 2014 | Ko *et al.*^78^ |
| 1980 | Holmes *et al.*^7^ | 1999 | Satoh *et al.*^43^ | 2014 | Kumar *et al.*^79^ |
| 1980 | Holmes *et al.*^8^ | 2000 | do Valle Chiossi *et al.*^44^ | 2014 | Minakawa *et al.*^80^ |
| 1980 | van de Wiel *et al.*^9^ | 2000 | Powell *et al.*^45^ | 2014 | Pérez *et al.*^81^ |
| 1981 | Eberst *et al.*^10^ | 2001 | Loo *et al.*^46^ | 2014 | Piva *et al.*^82^ |
| 1981 | Tindall *et al.*^11^ | 2002 | Amato *et al.*^47^ | 2014 | Samson *et al.*^83^ |
| 1982 | Burkhart^12^ | 2002 | Shimanovich *et al.*^48^ | 2014 | Senturk *et al.*^84^ |
| 1982 | Cutler^13^ | 2003 | Djahansouzi *et al.*^49^ | 2014 | Yang et al.^85^ |
| 1982 | Miyagawa *et al.*^14^ | 2003 | Wöhrl *et al.*^50^ | 2015 | Huilaja *et al.*^86^ |
| 1982 | Quimbly *et al.*^15^ | 2004 | Al-Mutairi *et al.*^51^ | 2015 | Nguyen *et al.*^87^ |
| 1982 | Slazinski *et al.*^16^ | 2004 | Kreuter *et al.*^52^ | 2015 | Seidel *et al.*^88^ |
| 1983 | Bercovitch *et al.*^17^ | 2004 | Wollina *et al.*^53^ | 2015 | Takayama *et al.*^89^ |
| 1983 | Shornick *et al.*^18^ | 2005 | Cozzani *et al.*^54^ | 2015 | Tani *et al.*^90^ |
| 1984 | Bonifazi *et al.*^19^ | 2005 | Heazell *et al.*^55^ | 2016 | Arakawa *et al.*^91^ |
| 1984 | MacDonald *et al.*^20^ | 2006 | Guven *et al.*^56^ | 2016 | Guerra *et al.*^92^ |
| 1984 | Shornick *et al.*^21^ | 2007 | Aoyama *et al.*^57^ | 2016 | Ingen-Housz-Oro *et al.*^93^ |
| 1985 | Fine *et al.*^22^ | 2007 | Cianchini *et al.*^58^ | 2016 | Özdemir *et al.*^94^ |
| 1986 | Costa *et al.*^23^ | 2007 | Rodrigues *et al.*^59^ | 2016 | Singla *et al.*^95^ |
| 1986 | Holmes *et al.*^24^ | 2007 | Hon *et al.*^60^ | 2017 | De la Calle *et al.*^96^ |
| 1987 | Jørgensen *et al.*^25^ | 2008 | Bedocs *et al.*^61^ | 2017 | Fania *et al.*^97^ |
| 1987 | Lao *et al.*^26^ | 2009 | Dahiya *et al.*^62^ | 2017 | Tourte *et al.*^98^ |
| 1990 | Eldor *et al.*^27^ | 2009 | Doiron *et al.*^63^ | 2017 | Zubor *et al.*^99^ |
| 1991 | Baxi *et al.*^28^ | 2010 | Lu *et al.*^64^ | 2018 | Almeida *et al.*^100^ |
| 1991 | Karna *et al.*^29^ | 2011 | Campbell *et al.*^65^ | 2018 | Dabas *et al.*^101^ |
| 1991 | Wolman *et al.*^30^ | 2011 | Marker *et al.*^66^ | 2018 | Veiga *et al.*^102^ |
| 1992 | Garvey *et al.*^31^ | 2012 | Fukuda *et al.*^67^ | 2018 | Oumerzouk *et al.*^103^ |
| 1994 | Kirtschig *et al.*^32^ | 2012 | Gan *et al.*^68^ | 2018 | Papapanagiotou *et al.*^104^ |
| 1994 | Naganathan *et al.*^33^ | 2012 | Patsatsi *et al.*^69^ | 2018 | Soares *et al.*^105^ |
| 1994 | Tanzi *et al.*^34^ | 2012 | Takatsuka *et al.*^70^ | 2018 | Warshafsky *et al.*^106^ |
| 1995 | Ibbotson *et al.*^35^ | 2012 | Thomas *et al.*^71^ | 2018 | Yang *et al.*^107^ |
| 1996 | Castle *et al.*^36^ | 2012 | Westermann *et al.*^72^ | 2019 | Merson *et al.*^108^ |
|  |  |  |  | 2019 | Wang *et al.*^109^ |

1. Hertz KC, Katz SI, Maize J, Ackerman AB. Herpes gestationis. A clinicopathologic study. *Arch Dermatol* 1976; **112**:1543‐1548.
2. Reid LH, Sams WM Jr. Letter: Herpes gestationis. *Arch Dermatol* 1976; **112**:882.
3. Carruthers JA, Black MM, Ramnarain N. Immunopathological studies in herpes gestationis. *Br J Dermatol 1977*; **96**:35‐43.
4. Reunala T, Karvonen J, Tiilikainen A, Salo OP. Herpes gestationis. A high titre of anti-HLA-B8 antibody in the mother and pemphigoid-like immunohistological findings in the mother and the child. *Br J Dermatol* 1977; **96**:563‐568.
5. Schiffner JH, Rea TH, Quismorio FP. Herpes gestationis. *Arch Dermatol* 1978; **114**:448.
6. Grimwood R, Arroyave CM, Weston WL, Aeling JL. Herpes gestationis associated with the C3 nephritic factor. *Arch Dermatol* 1980; **116**:1045‐1047.
7. Holmes R, Black MM, Williamson DM, Scutt RW. Herpes gestationis and bullous pemphigoid: a disease spectrum. *Br J Dermatol* 1980; **103**:535‐541.
8. Holmes RC, Black MM. Herpes gestationis. A possible association with autoimmune thyrotoxicosis (Graves' disease). *J Am Acad Dermatol* 1980; **3**:474‐477.
9. Van de Wiel A, Hart HC, Flinterman J, Kerckhaert JA, Du Boeuff JA, Imhof JW. Plasma exchange in herpes gestationis. *Br Med J* 1980; **281**:1041‐1042.
10. Eberst E, Tongio MM, Eberst B, Mayer S, Basset A. Herpes gestationis and anti-HLA immunization. *Br J Dermatol* 1981; **104**:553‐559.
11. Tindall JG, Rea TH, Shulman I, Quismorio FP Jr. Herpes gestationis in association with a hydatidiform mole. Immunopathologic studies. *Arch Dermatol* 1981; **117**:510‐512.
12. Burkhart CG. Pyridoxine-responsive herpes gestationis. *Arch Dermatol* 1982; **118**:535.
13. Cutler TP. Herpes gestationis. *Clin Exp Dermatol* 1982; **7**:201‐207.
14. Miyagawa S, Yoshioka J, Morita M, Sakamoto K. Herpes gestationis associated with ectopic pregnancy. *J Dermatol* 1982; **9**:203‐206.
15. Quimby SR, Xenias SJ, Perry HO. Herpes gestationis. *Mayo Clin Proc* 1982; **57**:520‐526.
16. Slazinski L, Degefu S. Herpes gestationis associated with choriocarcinoma. *Arch Dermatol* 1982; **118**:425‐428.
17. Bercovitch L, Bogaars HA, Murray DO. Pustular herpes gestationis. *Arch Dermatol* 1983; **119**:91‐93.
18. Shornick JK, Bangert JL, Freeman RG, Gilliam JN. Herpes gestationis: clinical and histologic features of twenty-eight cases. *J Am Acad Dermatol* 1983; **8**:214‐224.
19. Bonifazi E, Meneghini CL. Herpes gestationis with transient bullous lesions in the newborn. *Pediatr Dermatol* 1984; **1**:215‐218.
20. Macdonald KJ, Raffle EJ. Ritodrine therapy associated with remission of pemphigoid gestationis. *Br J Dermatol* 1984; **111**:630.
21. Shornick JK, Meek TJ, Nesbitt LT Jr, Gilliam JN. Herpes gestationis in blacks. *Arch Dermatol* 1984; **120**:511‐513.
22. Fine JD, Omura EF. Herpes gestationis. Persistent disease activity 11 years post partum. *Arch Dermatol* 1985; **121**:924‐926.
23. Costa C, Posternak FR. Ritodrine and pyridoxine in herpes gestationis. *Dermatologica* 1986; **173**:102‐103.
24. Holmes RC, Williamson DM, Black MM. Herpes gestationis persisting for 12 years post partum. *Arch Dermatol* 1986; **122**:375‐376.
25. Jørgensen EH, Clemmensen O, Anagnostaki L. Herpes gestationis. Acta Obstet Gynecol *Scand* 1987; **66**:175‐177.
26. Lao TT, Chang AM. An unusual presentation of herpes gestationis. *Asia Oceania J Obstet Gynaecol* 1987; **13**:283‐286.
27. Eldor J, Zlotogorski A, Meirow D, Cohen M. Epidural analgesia for a parturient with herpes gestationis. *Can J Anaesth* 1990; **37**:678‐679.
28. Baxi LV, Kovilam OP, Collins MH, Walther RR. Recurrent herpes gestationis with postpartum flare: a case report. *Am J Obstet Gynecol* 1991; **164**:778‐780.
29. Karna P, Broecker AH. Neonatal herpes gestationis. *J Pediatr* 1991; **119**:299‐301.
30. Wolman I, Ophir J, Pauzner D, Brenner S, David MP. Pemphigus gestationis (herpes gestationis): a case report. *Am J Obstet Gynecol* 1991; **165**:743‐744.
31. Garvey MP, Handfield-Jones SE, Black MM. Pemphigoid gestationis--response to chemical oophorectomy with goserelin. *Clin Exp Dermatol* 1992; **17**:443‐445.
32. Kirtschig G, Collier PM, Emmerson RW, Wojnarowska F. Severe case of pemphigoid gestationis with unusual target antigen. *Br J Dermatol* 1994; **131**:108‐111.
33. Naganathan K, Doi SA, Jamiyah H. Herpes gestationis: one of the rare skin lesions in pregnancy. *Med J Malaysia 1994*; **49**:401‐403.
34. Tanzi P, Lojacono A, Tarantini M, Faden D. Herpes gestationis. *Int J Gynaecol Obstet* 1994; **45**:47‐49.
35. Ibbotson SH, Lawrence CM. An uninvolved pregnancy in a patient after a previous episode of herpes gestationis. *Arch Dermatol* 1995; **131**:1091‐1092.
36. Castle SP, Mather-Mondrey M, Bennion S, David-Bajar K, Huff C. Chronic herpes gestationis and antiphospholipid antibody syndrome successfully treated with cyclophosphamide. *J Am Acad Dermatol 1996*; **34**:333‐336.
37. Jenkins RE, Jones SA, Black MM. Conversion of pemphigoid gestationis to bullous pemphigoid--two refractory cases highlighting this association. *Br J Dermatol* 1996; **135**:595‐598.
38. Lowe PM, Fryer J, Shumack S. Pemphigoid gestationis occurring in a patient with HELLP syndrome. Australas J Dermatol 1996; **37**:89‐92.
39. Schild RL, Owens OJ. Hereditary angioneurotic oedema in pregnancy coincidentally associated with herpes gestationis. *J Obstet Gynaecol* 1997; **17**:153.
40. Vaughan Jones SA, Bhogal BS, Black MM, Clement M, Hashimoto T, Nishikawa T. A typical case of pemphigoid gestationis with a unique pattern of intercellular immunofluorescence. *Br J Dermatol* 1997;**136**:245‐248.
41. Hern S, Harman K, Bhogal BS, Black MM. A severe persistent case of pemphigoid gestationis treated with intravenous immunoglobulins and cyclosporin. *Clin Exp Dermatol* 1998; **23**:185‐188.
42. Chen SH, Chopra K, Evans TY, Raimer SS, Levy ML, Tyring SK. Herpes gestationis in a mother and child. *J Am Acad Dermatol* 1999; **40**:847‐849.
43. Satoh S, Seishima M, Sawada Y, Izumi T, Yoneda K, Kitajima Y. The time course of the change in antibody titres in herpes gestationis. *Br J Dermatol* 1999; **140**:119‐123.
44. do Valle Chiossi MP, Costa RS, Ferreira Roselino AM. Titration of herpes gestationis factor fixing to C3 in pemphigoid herpes gestationis associated with choriocarcinoma. *Arch Dermatol* 2000; **136**:129‐130.
45. Powell J, Wojnarowska F, James M, Allott H. Pemphigoid gestationis with intra-uterine death associated with foetal cerebral haemorrhage in the mid-trimester. *Clin Exp Dermatol* 2000; **25**:452‐453.
46. Loo WJ, Dean D, Wojnarowska F. A severe persistent case of recurrent pemphigoid gestationis successfully treated with minocycline and nicotinamide. *Clin Exp Dermatol* 2001; **26**:726‐727.
47. Amato L, Coronella G, Berti S, Gallerani I, Moretti S, Fabbri P. Successful treatment with doxycycline and nicotinamide of two cases of persistent pemphigoid gestationis*. J Dermatolog* *Treat* 2002; **13**:143‐146.
48. Shimanovich I, Skrobek C, Rose C, et al. Pemphigoid gestationis with predominant involvement of oral mucous membranes and IgA autoantibodies targeting the C-terminus of BP180. *J Am Acad Dermatol* 2002; **47**:780‐784.
49. Djahansouzi S, Nestle-Kraemling C, Dall P, Bender HG, Hanstein B. Herpes gestationis may present itself as a paraneoplastic syndrome of choriocarcinoma-a case report. *Gynecol Oncol* 2003; **89**:334‐337.
50. Wöhrl S, Geusau A, Karlhofer F, Derfler K, Stingl G, Zillikens D. Pemphigoid gestationis: treatment with immunoapheresis. *J Dtsch Dermatol Ges* 2003; **1**:126‐130.
51. Al-Mutairi N, Sharma AK, Zaki A, El-Adawy E, Al-Sheltawy M, Nour-Eldin O. Maternal and neonatal pemphigoid gestationis. *Clin Exp Dermatol* 2004; **29**:202‐204.
52. Kreuter A, Harati A, Breuckmann F, Appelhans C, Altmeyer P. Intravenous immune globulin in the treatment of persistent pemphigoid gestationis. J Am Acad Dermatol 2004; **51**:1027‐1028.
53. Wollina U, Degen KW, Konrad H, Schönlebe J. Itching stretch marks and bullous lesions in a pregnant woman. *Int J Dermatol* 2004; **43**:752‐754.
54. Cozzani E, Basso M, Parodi A, Rebora A. Pemphigoid gestationis post partum after changing husband. *Int J Dermatol* 2005; **44**:1057‐1058.
55. Heazell AE, Sinha A, Bhatti NR. A case of gestational diabetes arising following treatment with glucocorticosteroids for pemphigoid gestationis. *J Matern Fetal Neonatal Med* 2005; **18**:353‐355.
56. Guven S, Erkin G, Tuncer ZS. Pemphigoid gestationis complicating a pregnancy following in vitro fertilization and intracytoplasmic sperm injection. *Int J Dermatol* 2006; **45**:1120‐1121.
57. Aoyama Y, Asai K, Hioki K, Funato M, Kondo N, Kitajima Y. Herpes gestationis in a mother and newborn: immunoclinical perspectives based on a weekly follow-up of the enzyme-linked immunosorbent assay index of a bullous pemphigoid antigen noncollagenous domain. *Arch Dermatol* 2007; **143**:1168‐1172.
58. Cianchini G, Masini C, Lupi F, Corona R, De Pità O, Puddu P. Severe persistent pemphigoid gestationis: long-term remission with rituximab. *Br J Dermatol* 2007; **157**:388‐389.
59. Rodrigues Cdos S, Filipe P, Solana Mdel M, Soares de Almeida L, Cirne de Castro J, Gomes MM. Persistent herpes gestationis treated with high-dose intravenous immunoglobulin. *Acta Derm Venereol* 2007; **87**:184‐186.
60. Hon KL, Chiu LS, Lam MC, Choi CL, Chan S, Luk NM. Normal neonatal outcome in a Chinese woman with pemphigoid gestationis, Graves' disease, and history of placental chorioangioma. *Int J Dermatol* 2007; **46**:996‐997.
61. Bedocs PM, Kumar V, Mahon MJ. Pemphigoid gestationis: a rare case and review. *Arch Gynecol Obstet* 2009; **279**:235‐238.
62. Dahiya K, Sharma D, Rajotia N, Jindal R. Herpes gestationis. *Taiwan J Obstet Gynecol* 2009; **48**:425‐427.
63. Doiron P, Pratt M. Antepartum intravenous immunoglobulin therapy in refractory pemphigoid gestationis: case report and literature review. *J Cutan Med Surg* 2010; **14**:189‐192.
64. Lu PD, Ralston J, Kamino H, Stein JA. Pemphigoid gestationis. *Dermatol Online J* 2010; **16**:10.
65. Campbell SM, Balazs K, Conroy M. Pemphigoid gestationis: a case report and review of the literature. *Cutis* 2011; **88**:21‐26.
66. Marker M, Derfler K, Monshi B, Rappersberger K. Successful immunoapheresis of bullous autoimmune diseases: pemphigus vulgaris and pemphigoid gestationis. *J Dtsch Dermatol Ges* 2011; **9**:27‐31.
67. Fukuda S, Ishii N, Hamada T, et al. A case of herpes gestationis: follow-up study of autoantibodies using enzyme-linked immunosorbent assay and immunoblotting. *Indian J Dermatol Venereol Leprol* 2012; **78**:199-201.
68. Gan DC, Welsh B, Webster M. Successful treatment of a severe persistent case of pemphigoid gestationis with antepartum and postpartum intravenous immunoglobulin followed by azathioprine. *Australas J Dermatol* 2012; **53**:66‐69.
69. Patsatsi A, Vavilis D, Tsikeloudi M, Kalabalikis D, Sotiriadis D. Refractory pemphigoid gestationis postpartum. *Acta Obstet Gynecol Scand* 2012; **91**:636‐637.
70. Takatsuka Y, Komine M, Ohtsuki M. Pemphigoid gestationis with a complete hydatidiform mole. *J Dermatol* 2012; **39**:474‐476.
71. Thomas S, Rajan U, George S, George M. Postpartum pemphigoid gestationis. *Indian J Dermatol* 2012; **57**:146‐148.
72. Westermann L, Hügel R, Meier M, et al. Glucocorticosteroid-resistant pemphigoid gestationis: successful treatment with adjuvant immunoadsorption. *J Dermatol* 2012; **39**:168‐171.
73. Matsumoto N, Osada M, Kaneko K, Ohara K, et al. Pemphigoid gestationis after spontaneous expulsion of a massive complete hydatidiform mole. *Case Rep Obstet Gynecol* 2013; **2013**:267268.
74. Podolec-Rubiś M, Wołek M, Brzewski P, Wojas-Pelc A. Suspicion of pulmonary embolism during treatment of pemphigoid gestationis. *Postepy Dermatol Alergol* 2013; **30**:59‐61.
75. Cabral R, Teixeira V, Brinca A, Fernandes B, Reis JP. Case for diagnosis. Pemphigoid gestationis. *An Bras Dermatol* 2014; **89**:167‐168.
76. Hapa A, Gurpinar A, Akan T, Gokoz O. A resistant case of pemphigus gestationis successfully treated with intravenous immunoglobulin plus cyclosporine. *Int J Dermatol* 2014; **53**:e269‐e271.
77. Henry S. Recognizing presentations of pemphigoid gestationis: a case study. *Case Rep Obstet Gynecol* 2014; **2014**:415163.
78. Ko BJ, Whang KU. Intravenous Immunoglobulin Therapy for Persistent Pemphigoid Gestationis with Steroid Induced Iatrogenic Cushing's Syndrome. *Ann Dermatol* 2014; **26**:661‐663.
79. Kumar S, Biswas M, Rajagopal R, Datta S, Narayan B. Successfully treated case of pemphigoid gestationis with post-partum intravenous and oral steroids. *J Obstet Gynaecol India* 2014;**64**:16‐18.
80. Minakawa S, Kaneko T, Rokunohe D, et al. Pemphigoid gestationis with prepartum flare. *J Dermatol* 2014; **41**:850-1.
81. Pérez J, Aspillaga S, Castro A, Clavería P, Sepúlveda R. Dyshidrosiform presentation (pompholyx-like) of pemphigoid gestationis with intrauterine fetal death. *Int J Dermatol* 2014; **53**:1383‐1385.
82. Piva I, Lo Monte G, Graziano A, Marci R. Herpes Gestationis after Ovodonation: Is Placenta the only Target of the Immune Reaction?. *J Clin Diagn Res* 2014; **8**:OD01‐OD2.
83. Samson JF, Thomas MM, Philip M, et al. Pemphigoid gestationis (herpes gestationis). *J Obstet Gynaecol India* 2014; **64**:36‐39.
84. Sentürk S, Dilek N, Tekin YB, Colak S, Gündoğdu B, Güven ES. Pemphigoid gestationis in a third trimester pregnancy. *Case Rep Obstet Gynecol* 2014; **2014**:127628.
85. Yang B, Wang C, Wu M, et al. A case of pemphigoid gestationis with concurrent IgG antibodies to BP180, BP230 and type VII collagen. *Australas J Dermatol* 2014; **55**:e15‐e18.
86. Huilaja L, Mäkikallio K, Hannula-Jouppi K, Väkevä L, Höök-Nikanne J, Tasanen K. Cyclosporine treatment in severe gestational pemphigoid. *Acta Derm Venereol* 2015; **95**:593‐595.
87. Nguyen T, Alraqum E, Razzaque Ahmed A. Positive clinical outcome with IVIg as monotherapy in recurrent pemphigoid gestationis. *Int Immunopharmacol* 2015; **26**:1‐3.
88. Seidel R, Lavi N, Chipps L. Pemphigoid Gestationis: A Case Report and Review of Management. *J Drugs Dermatol* 2015; **14**:904‐907.
89. Takayama N, Nakazono S, Kumagai J, et al. Pemphigoid gestationis with IgG autoantibodies to both the 120 kDa LAD-1 and the BP180 NC16a domain. *Eur J Dermatol* 2015; **25**:190-2.
90. Tani N, Kimura Y, Koga H, et al. Clinical and immunological profiles of 25 patients with pemphigoid gestationis. *Br J Dermatol* 2015; **172**:120‐129.
91. Arakawa M, Ohata C, Tsuruta D, et al. A severe and prolonged case of pemphigoid gestationis successfully treated with combination therapies. Br J Dermatol 2016; **174**:925-7.
92. Guerra L, Mazzanti C, Calabresi V, De Luca N, Zambruno G, Di Zenzo G. Pemphigoid Gestationis Complicating an Egg Donation Pregnancy. *Acta Derm Venereol* 2016; **96**:695‐696.
93. Ingen-Housz-Oro S, Sbidian E, Ortonne N, Poirier E, Chosidow O, Wolkenstein P. Pemphigoid gestationis revealing a denial of pregnancy. *J Eur Acad Dermatol Venereol* 2016; **30**:1411‐1413.
94. Özdemir Ö, Atalay CR, Asgarova V, Ilgin BU. A resistant case of pemphigus gestationis successfully treated with cyclosporine. *Interv Med Appl Sci* 2016; **8**:20‐22.
95. Singla A, Shree S, Mehta S. Pregnancy with Pemphigoid Gestationis: A Rare Entity. *J Clin Diagn Res* 2016; **10**:QD06-7.
96. De la Calle M, Vidaurrázaga C, Martinez N, González-Beato M, Antolín E, Bartha JL. Successful treatment of a severe early onset case of pemphigoid gestationis with intravenous immunoglobulin in a twin pregnancy conceived with in vitro fertilisation in a primigravida. *J Obstet Gynaecol* 2017; **37**:246‐247.
97. Fania L, Guerriero C, Ricci F, Gagliano MF, De Simone C. Herpes gestationis and oral contraceptive: Case report and review of the literature. *Dermatol Ther* 2017; **30**:10.1111/dth.12518.
98. Tourte M, Brunet-Possenti F, Mignot S, Gavard L, Descamps V. Pemphigoid gestationis: a successful preventive treatment by rituximab. *J Eur Acad Dermatol Venereol* 2017; **31**:e206‐e207.
99. Zubor P, Svecova I, Moricova P, Pec J, Adamicova K, Danko J. Early flare-up of severe Herpes gestationis (Pemphigoid gestationis) and successfull prolonged treatment: A case report. *J Obstet Gynaecol* 2017; **37**:824‐826.
100. Almeida FT, Sarabando R, Pardal J, Brito C. Pemphigoid gestationis successfully treated with intravenous immunoglobulin. *BMJ Case Rep* 2018; **2018**:bcr2018224346.
101. Dabas G, Afra TP, De D, et al. Anhydramnios in Patients With Pemphigoid Gestationis. *JAMA Dermatol* 2018; **154**:484-486.
102. Veiga VF, Santos F, Antunes A, Duarte I. Phemphigoid gestationis. *BMJ Case Rep* 2018; **2018**:bcr2018225242.
103. Oumerzouk J, Abida N, Zaimi A, Znati K, Zbir EM, Bourazza A. Severe pemphigoid gestationis associated with acute disseminated encephalomyelitis in the setting of a systemic disorder. *Australas J Dermatol* 2018; **59**:e123‐e126.
104. Papapanagiotou IK, Tsagouri S, Liakou CG, et al. Pemphigoid gestationis. *Clin Case Rep* 2018; **6**:1364-1365.
105. Soares KS, Lehmann PM, Hofmann SC. Pemphigoid gestationis with lethal fetal malformation and postpartum persistence. *J Dtsch Dermatol Ges* 2018; **16**:775-777.
106. Warshafsky C, Tron VA, Robertson D, Kives S. Pemphigoid Gestationis: A Case Presentation. *J Cutan Med Surg* 2018; **22**:435‐438.
107. Yang A, Uhlenhake E, Murrell DF. Pemphigoid gestationis and intravenous immunoglobulin therapy. *Int J Womens Dermatol* 2018; **4**:166-169.
108. Merson J, Murphy DC. What is causing this pregnant patient's rash? *JAAPA* 2019; **32**:54-56.
109. Wang L, Huang X, Wang M, Li L, Zeng K. Successful treatment of one case of pemphigoid gestationis complicating with polycystic ovary syndrome. *Dermatol Ther* 2019; **32**:e12898.

**Supplementary table 3**. Retrospective series excluded due to insufficient data about pemphigoid gestationis treatment

| **Authors** | **Total number of cases** | **Topical corticosteroids as monotherapy, n (%)** | **Systemic corticosteroids, n (%)** | **Systemic corticosteroids + steroid-sparing treatments, n (%)** | **Cited steroid-sparing treatments** |
| --- | --- | --- | --- | --- | --- |
| **Jenkins et al.** | 69 | 13 | 39 | 17 | Dapsone, pyridoxine, azathioprine, sulfapyridine, plasmapheresis |
| **Boulinguez et al. *** | 10 | NA | 2 | 2 | Azathioprine, cyclophosphamide |
| **Castro et al.** | 10 | 3 | 3 | 4 | Azathioprine, tetracycline |
| **Chi et al.** | 61 | NA | 39 | NA | None |
| **Al-Saif et al.** | 31 | 0 | 30 | 1 | Intravenous immunoglobulin therapy |
| **Cobo et al.** | 7 | 1 | 5 | 1 | Azathioprine |
| **Hallaji et al.** | 23 | 4 | 18 | 1 | Azathioprine, mycophenolate, rituximab |
| **Rassai et al.** | 13 | 1 | 12 | 0 | None |

NA, No data available

* no data on therapies for patients without persistent course (n=4); lost to follow-up (n=1); several associated therapies not specified (n=1)

Al-Saif F, Elisa A, Al-Homidy A, Al-Ageel A, Al-Mubarak M. Retrospective analysis of pemphigoid gestationis in 32 Saudi patients - Clinicopathological features and a literature review. *J Reprod Immunol* 2016; **116**:42‐45.

Boulinguez S, Bédane C, Prost C, Bernard P, Labbé L, Bonnetblanc JM. Chronic pemphigoid gestationis: comparative clinical and immunopathological study of 10 patients. *Dermatology* 2003; **206**:113‐119.

Castro LA, Lundell RB, Krause PK, Gibson LE. Clinical experience in pemphigoid gestationis: report of 10 cases. *J Am Acad Dermatol* 2006; **55**:823‐828.

Chi CC, Wang SH, Charles-Holmes R, et al. Pemphigoid gestationis: early onset and blister formation are associated with adverse pregnancy outcomes. *Br J Dermatol* 2009; **160**:1222‐1228.

Cobo MF, Santi CG, Maruta CW, Aoki V. Pemphigoid gestationis: clinical and laboratory evaluation. *Clinics (Sao Paulo)* 2009; **64**:1043‐1047.

Hallaji Z, Mortazavi H, Ashtari S, Nikoo A, Abdollahi M, Nasimi M. Pemphigoid gestationis: Clinical and histologic features of twenty-three patients. *Int J Womens Dermatol* 2016;**3**:86‐90.

Jenkins RE, Hern S, Black MM. Clinical features and management of 87 patients with pemphigoid gestationis. *Clin Exp Dermatol* 1999; **24**:255‐259.

Rassai S, Pazyar N, Alavi SM, Kazerouni A, Feily A. Pemphigoid gestationis: a retrospective study in southwest Iran. *Acta Med Iran* 2013; **51**:408-10.
